# Texas Carries off the Honors

**Published:** 1886-03

**Authors:** 


TEXAS CARRIES OFF THE HONORS. (AS USUAL).

We are pleased to note in the *Southern Practitioner* (Nashville), in an editorial account of the commencement exercises of the Medical and Dental Departments of the University of Tennessee, the names of two Texas students who won first honors, to-wit, Dr. R. W. Freeman, valedictorian, and Dr. E. G. Stuart, faculty medal for excellence in all branches—first prize ; and moreover the *Practitioner* pays one of the gentlemen, Dr. Freeman, the valedictorian, the compliment to say :

" The delivery of Dr. Freeman's address was listened to with the most wrapped attention shown by any audience that ever occupied the halls, notwithstanding that its halls have reverberated, echoed, and resounded to the tones of Edwin Forest, Edmund Kean, McCullough, Jefferson, and others of the best readers of the English language."

We have not the honor of the acquaintance of either Dr. Freeman or Dr. Stuart, nor do we know their post office address ; but we extend to them our hearty congratulation on the handsome manner in which they have "held up" the Texas corner, and sustained her reputation—for this thing of first honors to Texas students is getting to be of yearly occurrence ; and we extend to them the right hand, and bid them welcome to the ranks of honorable medicine. We look to see them adorn the profession they have chosen, and will watch their career with interest. Hurrah for Texas!

